# LCSS-Based Algorithm for Computing Multivariate Data Set Similarity: A Case Study of Real-Time WSN Data

**DOI:** 10.3390/s19010166

**Published:** 2019-01-04

**Authors:** Rahim Khan, Ihsan Ali, Saleh M. Altowaijri, Muhammad Zakarya, Atiq Ur Rahman, Ismail Ahmedy, Anwar Khan, Abdullah Gani

**Affiliations:** 1 Department of Computer Science, Abdul Wali Khan University, Mardan 23200, Pakistan; mohd.zakarya@awkum.edu.pk; 2 Department of Computer System and Technology, Faculty of Computer Science and IT, University of Malaya, Kuala Lumpur 50603, Malaysia; ismailahmedy@um.edu.my; 3 Faculty of Computing and Information Technology, Northern Border University, Rafha 91911, Saudi Arabia; Saltowaijri@nbu.edu.sa (S.M.A.); atiq621@gmail.com (A.U.R.); 4 Department of Electronics, University of Peshawar, Peshawar 25000, Pakistan; arkhan@uop.edu.pk; 5 School of Computing and Information Technology, Taylor’s University, Subang Jaya 47500, Malaysia

**Keywords:** multivariate data set, longest common subsequence, dynamic programming, WSN data

## Abstract

Multivariate data sets are common in various application areas, such as wireless sensor networks (WSNs) and DNA analysis. A robust mechanism is required to compute their similarity indexes regardless of the environment and problem domain. This study describes the usefulness of a non-metric-based approach (i.e., longest common subsequence) in computing similarity indexes. Several non-metric-based algorithms are available in the literature, the most robust and reliable one is the dynamic programming-based technique. However, dynamic programming-based techniques are considered inefficient, particularly in the context of multivariate data sets. Furthermore, the classical approaches are not powerful enough in scenarios with multivariate data sets, sensor data or when the similarity indexes are extremely high or low. To address this issue, we propose an efficient algorithm to measure the similarity indexes of multivariate data sets using a non-metric-based methodology. The proposed algorithm performs exceptionally well on numerous multivariate data sets compared with the classical dynamic programming-based algorithms. The performance of the algorithms is evaluated on the basis of several benchmark data sets and a dynamic multivariate data set, which is obtained from a WSN deployed in the Ghulam Ishaq Khan (GIK) Institute of Engineering Sciences and Technology. Our evaluation suggests that the proposed algorithm can be approximately 39.9% more efficient than its counterparts for various data sets in terms of computational time.

## 1. Introduction

Multivariate data set similarity is an emerging area of research because such data sets are generated routinely in scientific experiments, industries, educational organizations, on the web and in databases [1]. This problem exists in different applications, such as wireless sensor networks (WSNs), DNA comparison, computational biology, pattern matching, agriculture and file comparison. In the literature, various methodologies have been proposed to compute similarity indexes, among which the most prominent approach is the classical non-metric-based mechanism called longest common subsequence [2,3].

A data set is defined as the collection of values or numbers related to a particular object or entity. In computer science, it corresponds to a single table of the entire database and is represented by $X_{m,n}$ where *m* and *n* represent rows and columns, respectively. The subset of $X_{m,n}$ is obtained by removing zero or more rows or columns from *X*. The rows of a data set correspond to a sequence denoted by $A_{i}$ where i=1,…,n. A subsequence is obtained by removing zero or more numbers from *A*. Two sequences $A_{i}$ and $B_{j}$ are considered similar if their similarity index is higher than the defined threshold value. Similarly, for two data sets $X_{i,j}$ and $Y_{k,l}$, the problem of Longest Common Subsequence (LCSS) is computing their common subsequence, which has the longest length among all common subsequences of *X* and *Y* [4,5].

The most prominent approach is the traditional dynamic programming-based LCSS algorithm that does exceptionally well in computing similarity indexes of sequences or univariate data sets, but does not correctly solve the problem of multivariate data set similarity indexes [6]. In the literature, this algorithm has been extended in different ways to improve its efficiency and accuracy to solve multivariate problems; however, these algorithms are either application specific or exhibit complexity, scalability and dimensionality issues. All common subsequence algorithms have tried to address the issue associated with traditional dynamic programming-based algorithms and described the importance of second- and third-level common subsequences [7].

**Figure 1:**
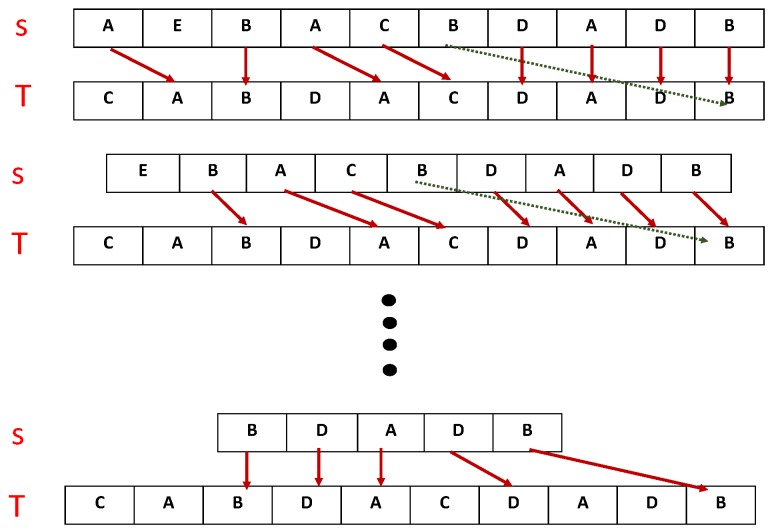
An overview of the proposed similarity computation mechanism.

Dynamic Time Warping (DTW) approaches the problem by stretching the multivariate data sets with respect to time [8]. In addition, Principal Component Analysis (PCA)-based techniques have tried to overcome this problem by avoiding unnecessary components of the data sets [9]. A tightly coupled issue associated with traditional dynamic programming based algorithms is their time and space complexities. These algorithms match every element of sequence S with each element of sequence T as shown in Figure 1, i.e., matching first element, that is A, in S with last element of T, that is B, which is unrealistic. These comparisons are necessary for the creation of a similarity indexes table that is used to compute the longest common subsequence (LCSS). Additionally, if two sequences are exactly the same, dynamic programming based algorithms will still compute their similarity indexes table. Likewise, the major issues associated with DTW and PCA-based techniques are their sensitivity to outliers and information loss, respectively. Therefore, a robust algorithm is needed to solve multivariate problems and avoid the issues of existing algorithms, such as outliers, scalability and complexity. In this study, we present an efficient algorithm to measure the similarity indexes of multivariate time-series data sets using a non-metric based approach, which is preferred due to its non-sensitive nature to outliers, as described in Figure 1, where σ=3 in this case. The proposed algorithm works by dividing the multivariate data sets into discrete univariate data sets and initiates the similarity indexes computation process with the first i.e., first column of multivariate data sets, as described in Figure 1, where σ=3. In this case, every element of data set S is matched with at most three elements of T and the matched elements, as depicted by red arrows, are stored with their location information. Additionally, the proposed algorithm uses sliding window control parameter (σ) to bound or limit the matching space of data set-1 elements in data set-2 which saves both time and space by avoiding unnecessary comparisons as depicted by the green arrow (as an example). Our experimental results show that the proposed algorithm solves most of the issues associated with the aforementioned dynamic programming-based algorithms. The remainder of the paper is organised as follows.

A brief overview of the literature is presented in the subsequent Section 2. Section 3 identifies the problem and elaborates the preprocessing procedure for the data sets. Section 4 presents the proposed algorithm and its working mechanism. This section also describes the mathematical model or formulation for the proposed approach. Section 5 describes the complexity and results of the proposed algorithm and several existing field-proven algorithms using real and benchmark data sets. Finally, Section 6 concludes this study and describes several future research directions.

## 2. Background and Previous Work

Multivariate time series is generated routinely in engineering, scientific, medical, academic, stock market, multimedia and industrial domains [10]. These data sets are difficult to investigate and their similarity indexes are difficult to compute using existing techniques due to their multivariate nature. Some of the closely related existing multivariate data set algorithms are described as follows. In 2003, Chiu et al. [11] successfully used Euclidean distance measure for retrieving one-dimensional motifs from multivariate time series. Mueen et al. [12] extended the idea of one-dimensional motif in 2009. However, this method is sensitive to noise and its complexity is directly proportional to the size and dimension of data sets. Xun et al. [13] presented a new approach for measuring the similarity of multivariate time series by considering the external structure of the matrix and the correlation between columns. Benson et al. [14] and Deorowicz et al. [15] presented LCSS approaches based on the dynamic programming concept in *k*-length sub-string problems. The performance of these algorithms is exceptional on small data sets, which degrade drastically within data set size and the value of *k*. In [16] the authors tried to address the open challenge that the detection rate decreases significantly when the number of features and training samples increases intrusions in WSN data traffic. They have proposed an efficient Intrusion Detection System (IDS) based on hybrid heuristic optimization algorithm, which is inspired by magnetic field theory in physics that deals with attraction between particles scattered in the search space. The developed algorithm helped in extracting the most relevant features that can assist in accurately detecting the network attacks. Another attempt to analyze WSN’s data set was presented by Yohei et al. [17] to perform a study using LCSS in at least *k*-length order-isomorphic sub-strings.

An alternative approach called PCA has been used in reducing the dimensionality of multivariate time series of data sets to reduce the computation cost and time. The data set dimensionality is reduced to preserve the overall energy or information. By applying a single-value transformation mechanism, the original multivariate time series is initially transformed, and the best coefficients are then selected to represent the original time series [18]. In 2001, Keogh et al. [19] presented a PCA-based technique for measuring similarity between multivariate time-series data sets. Traditionally, PCA-based methods are used as a preprocessing phase to identify correlated columns of multivariate data sets, and another distance measure is then used to find similarity of the transformed data. In 2004, Young et al. [20] proposed a mechanism to measure the similarity indexes of multivariate time series of data sets by combining the features of extended Frobenius norm error and PCA method; although the method worked deliberately well on different data sets, careful attention must be considered in handling the complexity issue. A robust PCA algorithm was proposed in [21] to overcome some of the major issues associated with this method. Although this algorithm works well, several limitations still exist, such as information loss, additional processing time (overheads) and limited applications or data sets, which should be considered for improvement.

In 2002, Vlachos et al. [22] proposed a mechanism to retrieve object trajectories up to three-dimensional spaces from multivariate data sets, which were then analysed to determine their similarity indexes. Trajectory classification is automatically conducted by using the k-nearest neighbour classification technique, and the non-metric-based approach, namely, LCSS, is then used to find the similarity between multidimensional trajectories. The complexity of extending this model to two or more dimensions is relatively high, which renders handling parallel movements difficult. In 2004, Duchene et al. [23] presented a similarity measure for multivariate time series, which is an extension of the non-metric-based algorithm proposed in [24]. This measure is applicable for heterogeneous parameters of multivariate time series and is efficient, particularly when time constraints are small.

In 2005, Sakurai et al. [25] proposed a mechanism for finding similarity between multivariate data sets based on the well-known DTW algorithm. DTW allows the squeezing and stretching of time series along the time-axis and imposes certain restrictions on warping paths, such as (i) points should be monotonically ordered; (ii) continuity; (iii) warping windows; (iv) slope constraints; and (v) boundary conditions. Initially, DTW was used for speech recognition to remove the shortcomings introduced by inflexibility along the time-axis. Thereafter, it was used for computing similarity between multivariate time-series data. Keogh et al. [26] proposed another DTW-based approach in 2012 to compute sequence similarity but failed to mention its performance and efficiency for multivariate data sets. Gorecki et al. [27] proposed a classification technique for multivariate data sets that is based on the idea of dynamic time warping windows. Initially, the algorithm calculates two distance measures, that is dynamic time warping distance between multivariate data sets and their derivatives. These distance measures are used with nearest neighbor rule to form a robust classification mechanism for multivariate data sets, that is easily adaptable in different application scenarios i.e., data sets. However, computational time complexity is still a challenging task and an open research problem for these algorithms, particularly the dynamic time warping based approach. Shojafar et al. [28] suggested a bandwidth and delay efficient data searching technique for hybrid infrastructure (i.e., ad hoc, WSN and fog computing). The proposed method utilized local information at fog node to accelerate the searching process. However, the results were limited to numerical evaluation and must be tested on real-time or benchmark data sets. The computational complexity of DTW is quadratic in time series length. Outliers are also a problem for DTW.

Numerous studies have been conducted on similarity approaches for sequence data, but few for multivariate data sets due to their applications in different areas. Therefore, this problem must be given considerable attention and a robust algorithm should be developed. The major problems associated with existing algorithms are their complexities, computational time, space, information loss and outliers/noise. Thus, an algorithm that can cope with these challenges to solve most of these issues is necessary.

## 3. Proposed Technique: An Overview

In the literature [15,17], LCSS algorithms are mostly based on the dynamic programming concept that compares every symbol of a time series/data set/sequence with every symbol of another to construct a similarity matrix of size m×n. This matrix is used to find the LCSS that will determine the required similarity indexes. LCSS algorithms are exceptionally good for univariate time series but possess extremely high time and space complexities for multivariate data sets. To overcome these issues, a robust algorithm is presented in this study to compute multivariate LCSS.

### 3.1. Preprocessing Phase

Time series is a sequence of observations generated simultaneously, and different time series have different range of values. For example, in stock exchange, the prices in January and December may range from PKR 30 million to 40 million and from PKR 60 million to 70 million, respectively. Therefore, data sets should be normalised before their analysis for similarity indexes or other processing. In the literature [29], various normalisation techniques exist; however, for simplicity, the technique adopted in this paper is represented by the following Equation (1):

(1) $$T_{i}=\frac{T_{i}-T_{min}}{T_{max}-T_{min}},$$

where $T_{i}$ represents the time series; and $T_{max}$ and $T_{min}$ denote the maximum and minimum values in *T*, respectively.

### 3.2. Proposed Mechanism

The proposed approach calculates LCSS by matching the first symbol of time series with every symbol of the second time series until a match occurs or a sliding window control parameter (σ) ends it. In the case of matched symbols, elements are stored with their location information. Similarly, the mechanism continues by matching the second symbol of the first time series with the symbols of the second time series starting from the previously matched location. This procedure is repeatedly applied to the remaining symbols of the first time series until the desired LCSS is calculated or the time series ends. Consider two univariate time series S=AEBACFDADB and T=CABDACDADB as shown in Figure 1. The first symbol *A* from *S* is matched with every symbol of *T* until a match is found, that is, the second location in *T*. Symbol *A* is stored along with its location information. Now, the second symbol *E* from *S* is compared with symbols of *T* starting from a previously matched location, that is, the third symbol. Additionally, this algorithm uses a sliding window control parameter (σ) to avoid unnecessary comparisons. This procedure is applied to the remaining symbols of *T* and *S* until their LCSS is computed, which is ABACDADB.

## 4. Proposed LCSS Calculation Mechanism

LCSS is a mechanism widely used to determine the similarity of data sets. Traditional dynamic programming-based algorithms are the most prominent way of describing the degree of similarity between two data sets. However, this method does not consider the properties of the data sets; in particular, whether the similarity index is 100 or 0, the procedure is the same. The method also does not consider the space required by comparing the first symbol of a data set with the last symbol of another data set, and the handling of multivariate data sets is relatively complex. Therefore, an efficient algorithm is necessary to overcome these diverse problems. To resolve these issues, a robust and efficient multivariate data set similarity-measuring algorithm is presented in the following Section 4.1.

### 4.1. Proposed Sequential Approach for Multivariate Time Series: Real-Time Data Set of WSN

Consider the multivariate data sets given in Table 1 and Table 2, which are a subset of the data set generated by the WSN deployed in the GIK Institute’s Orange Orchard (http://www.giki.edu.pk), as described in [30]. Wasp-mote agriculture boards, which comprise complete units with four sensors and a transceiver module, are deployed at different locations. These boards continuously collect soil moisture, air temperature, air humidity and leaf wetness values after a pre-defined time interval of 30 min. This frequency of data collection indicates that the boards generate 48 packets per day and night, 1440 packets per month and 17,520 packets per year. The distances between boards and the gateway are adjusted according to their wireless communication range of 500 m in the Xbee module, and the sensed data are transmitted by these nodes to a central location, specifically to a computer at a remote location, through the gateway module. The proposed technology called assisted decision support system, as demonstrated in [30], is deployed in a real agriculture environment for about one year to generate a real-time multivariate data set of approximately 52,000 records, each composed of a five-tuple or five variables.

**Figure 2:**
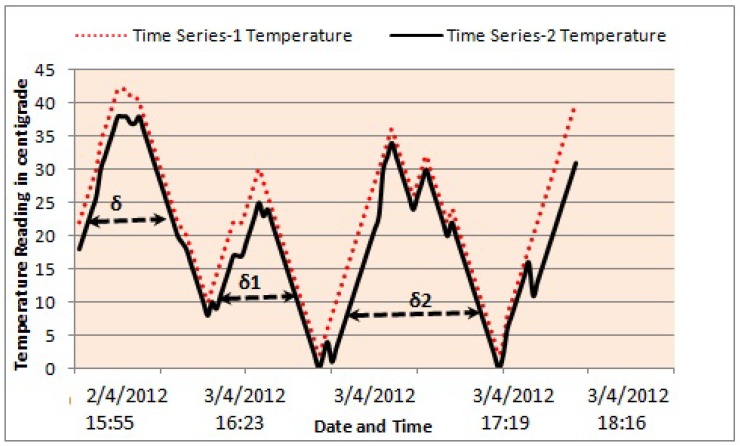
Sliding window-based control parameter to reduce the total number of comparisons needed to find a match in both data sets. Here, the *x*-axis shows the time of the collected temperature data, and the *y*-axis shows the value of the data in ${}^{\circ }$C.

Every variable of a multivariate time series is treated as a univariate time series, that is, temperature, humidity and moisture represent different time series. The proposed algorithm works by finding the LCSS of the first variable, which is temperature in this case. The first temperature value of Table 1 (i.e., 35 ${}^{\circ }$C) is matched with every temperature value of Table 2 until a match is found or σ ends it or the end of the data sets are reached. Please note that σ is a sliding window control parameter that defines a specific window (i.e., a range of values to be considered for matching) for each element of time series/data set 1 (Table 1) in time series/data set 2 (Table 2), which is three in this case. The sliding window control parameter is an important aspect to be considered because matching of the first element/symbol of a sequence/time series/data set with the last element of another does not make any sense at all due to the fact that the problem under consideration requires finding the LCSS. The idea of the sliding window control parameter is described in Figure 2, where different values of this parameter are given and their selection from σ, $\sigma_{1}$ and $\sigma_{2}$ depends on the application and data set. A σ with a smaller value is selected if abrupt variations or fluctuations in data sets are occasionally encountered i.e., temperature reading collected through a sensor node. Conversely, a larger σ value is an ideal candidate or solution for multivariate data sets which have an extremely high variation or fluctuation ratio i.e., stock exchange data. The control parameter is helpful in reducing the total number of comparisons, in this case temperature value 35 ${}^{\circ }$C (Table 1), needed to find the matching element in Table 2. Traditional dynamic programming-based algorithms (as described in Section 2) compare a single value to all values of the second data set, which can be expensive or even inappropriate in real time. Our sliding window-based comparison technique, as described in Figure 2, limits the matching window to a particular value (i.e., the first value of Table 1 is matched with only three values of Table 2).

In situations where a match is encountered, elements are stored with location information or time stamps (i.e., 35 ${}^{\circ }$C at time $T_{1}$ in *S* and time $T_{2}$ in *T*). Similarly, the second element of *S*, that is, 39 ${}^{\circ }$C, is matched with every element of *T* starting from the previously stored matched location, which is 36 ${}^{\circ }$C. As no match for this element is found in the allowable matching window, the next element is considered for matching, that is, 36 ${}^{\circ }$C is matched with the third element of *T*. The value 36 ${}^{\circ }$C is stored with its time stamp information, and the next element is taken. This procedure is repeated for the remaining elements/symbols of *S*, and a common subsequence with its length information is produced. The next step is removal of the first element from *S*. The aforementioned procedure is repeated for the remaining elements until the LCSS for temperature only is found or the 1/4th or the mid-element of the time series is encountered. The length of the LCSS for variable 1 (i.e., temperature) is 8.

After successful calculation of the LCSS for the first variable, the next step is to find the joint LCSS of variables 1 and 2, which are temperature and humidity in this case, respectively. This procedure is relatively simple because all of the information required to match the elements of *S* and *T* at the location/time stamps recorded are already stored. For example, in a given time series, the similarity check is performed at the eight different time stamps where the temperature values are matched/similar. If the symbols are found equal, then the value of a particular time stamp is retained and updated with the humidity value; otherwise, the non-matched value with its corresponding time stamp is deleted. The humidity value of *S* at time stamp 15 April 2011 0:00 of Table 1 (i.e., 82% RH) and of *T* at time stamp 16 April 2011 0:00 (i.e., 81% RH) do not match. Therefore, this time stamp is deleted. This procedure is repeated for the remaining values/symbols/elements and reduces the LCSS from a length of 8 to 7. The procedure is applied repeatedly for the remaining variables until the multivariate LCSS is calculated.

### 4.2. Proposed Approach Mathematical Background

Definition of basic terms is given below.

Time series $a_{v_{1},t}$, $a_{v_{2},t}$, $a_{v_{3},t}$, …, $a_{v_{n},t}$ is represented by $A_{i,v,t}$ where *t* represents time, *v* number of variables and $a_{i}$ represent values. The second time series is represented by $B_{j,v,t}$. The longest common subsequence is represented by $LCS(m_{i,j,v})$ where $m_{i,j,v}$ do contain LCSS length and matched location information. Moreover, concatenation of two symbols/elements is written as XY where *X* and *Y* are symbols.

**Definition** **1.** *The LCS($m_{0})$ describes an LCSS of length zero.*

**Definition** **2.** *The $LCS(m_{i,j,v})$ is the LCSS of if $a_{iv}=b_{jv}$ and there exist $i_{{}^{\prime }}<i$ and $j_{{}^{\prime }}<j$ that generates $LCS(m_{i_{{}^{\prime }},j_{{}^{\prime }},v_{{}^{\prime }}})$.*

**Lemma** **1.** *For k>=1, $LCS(m_{i,j,v})$ is the LCSS iff $a_{iv}=b_{jv}$ and $length(LCS\left(m_{i,j,v}\right))>=k$. Thus the LCSS of $A_{i,v,t}$ and $B_{j,v,t}$ is of length k.*

**Proof.**  Applying mathematical induction on *k*. The $length(LCS\left(m_{1,1,v}\right))$ is equal to 1 iff $a_{1}=b_{1}$ (According to Definition 2), that produces/calculates LCSS of length 1. Hence, the lemma is true for k=1. Assume that it holds true for k−1 and needs to be proved for value of *k*. If $LCS(m_{i,j,v})$ is the LCSS of $A_{iv,t}$ and $B_{jv,t}$ then there exist $i^{{}^{\prime }}<i$ and $j^{{}^{\prime }}<j$ such that $LCS(m_{i^{{}^{\prime }},j^{{}^{\prime }},v^{{}^{\prime }}})$ is the LCSS of $A_{i^{{}^{\prime }}v,t}$ and $B_{j^{{}^{\prime }}v,t}$ for value of k−1. □

According to our assumptions, $LCS\left(m_{i^{{}^{\prime }},j^{{}^{\prime }},v^{{}^{\prime }}}\right)=c_{1vt},c_{2vt},c_{2vt}\ldots c_{i^{{}^{\prime }}vt}$ of time series $A_{i_{{}^{\prime }}v,t}$ and $B_{j_{{}^{\prime }}v,t}$ because $a_{i}=b_{j}$. Therefore, the LCSS of $A_{iv,t}$ and $B_{jv,t}$ is of length *k* because $length(LCS\left(m_{i^{{}^{\prime }},j^{{}^{\prime }},v^{{}^{\prime }}}\right)+m_{iv,jv})=k$. Hence, it proves that $length(LCS\left(m_{i,j,v}\right))>=k$.

Conversely, if $length(LCS\left(m_{i,j,v}\right))>=k$ and $a_{i}=b_{j}$ then there exist $i^{{}^{\prime }}<i$ and $j^{{}^{\prime }}<j$ such that $a_{i^{{}^{\prime }}}=b_{j^{{}^{\prime }}}$ and $length\left(LCS\left(m_{i^{{}^{\prime }},j^{{}^{\prime }},v^{{}^{\prime }}}\right)\right)=length\left(LCS\left(m_{i,j,v}\right)\right)-1>=k-1$. $LCS(m_{i^{{}^{\prime }},j^{{}^{\prime }},v^{{}^{\prime }}})$ is the LCSS of k−1 length (By inductive hypothesis). Hence, $LCS(m_{i,j,v})$ is the desired LCSS.

### 4.3. Proposed Multivariate LCSS Algorithm

Variable MD represents middle of time series and TP is used to store the position of matched elements. Length of the computed LCSS is stored in variable $Count_{1}$ and σ is used to defined the matching windows length for each symbol. LCSS is a multidimensional array used to store values of the computed LCSS and variable Count is used to identify accurate LCSS. The proposed multivariate data set similarity-measuring algorithm is presented below (Algorithm 1).

**Algorithm 1** Multivariate LCSS Calculation Procedure.**Require:** Multivariate data sets similarity **Ensure:** Return Longest Common Subsequence (LCSS)  1:LCSS(S,T)  2:Pre-LCSS ← 0  3:Cur-LCSS ← 0  4:TP ← 0  5:count ← 0  6:$count_{1}$← 0  7:for a ← 0 to M and $count_{1}<M$ and Dim is 1 do  8:   for i ← a to $S(Var_{1})$ do  9:      for j ← TP to $T(Var_{1})$ and σ<threshold−value do10:         if S or T = 0 then11:            LCSS ← 012:         elseif $S\left(Var_{1_{i}}\right)=T\left(Var_{1_{j}}\right)$ then13:            class $K_{1}$ ← *i*14:            class $K_{2}$ ← *j*15:            TP ←j+116:            count ←count+117:            break18:         elseif $S\left(Var_{1_{i}}\right)\neq T\left(Var_{1_{j}}\right)$ then19:            next element20:         end if21:      end for22:   end for23:   if count > $count_{1}$ then24:      Pre-LCSS ← Cur-LCSS25:      $count_{1}$← count26:      count ← 027:   end if28:   delete $a^{th}$ element from *S*29:   TP ← 030:end for31:for b ← 2 to *n* and *c*← element of class $K_{1}$ and *d*← elements of class $K_{2}$ do32:   if $S\left(Var_{b_{c}}\right)\neq T\left(Var_{b_{d}}\right)$ then33:      delete *c* and *d* elements from classes $K_{1}$ and $K_{2}$, respectively34:   end if35:end for36:**return** LCSS of data sets *S* and *T*


## 5. Complexity Analysis and Results Discussion

The performance and reliability of an algorithm is directly proportional to its complexity analysis and evaluation on benchmark data sets. In addition, every algorithm must be tested against field-proven algorithms under similar circumstances and data sets.

### 5.1. Proposed Algorithm Complexity Analysis

Complexity analysis is an important measure used to determine the reliability, applicability and robustness of an algorithm [1]. The best-case complexities of the proposed sequential algorithm and dynamic programming-based algorithms are **O**(**m** + **h**) and **O**(**m** × **n** + **h**), respectively, where *m* and *n* represent the length of the data sets, and *h* represents their dimensions or variables. The proposed algorithm is ideal in this situation because the LCSS is computed in one iteration, whereas the traditional LCSS algorithm will generate a similarity matrix and then compute for the LCSS. Similarly, the proposed sequential algorithm worst-case complexity is **O**(**M** × **n** × σ) + **O**(**h**), where σ is a constant, and M is the half-length of the time series. In the worst-case scenario, the performance of the proposed algorithm is better than that of dynamic programming-based algorithms because of its limited comparing window size.

To find the average case complexity, the problem is divided into disjoint sets of sub-problems and solved by starting from the simplest module to the most complex. The steps required to solve a particular problem range from j=0 to j=n. Some problems are solved by negligible work while others require a large number of steps (i.e., *n*). Assume that the probability of a subset taking *j* steps is represented by $p_{j}$. Then, the average case complexity is given by:

(2) $$T_{avg}\left(n\right)=\sum_{j=0}^{m}j\times p_{j}.$$

Let the probability of matched symbol and not matched symbol, in time series *S* and *T*, be given by Equations (3) and (4), respectively.

(3) $$P_{mtd}=\delta ,$$

(4) $$P_{nmtd}=\frac{1}{\delta }.$$

Their total probability of $P_{1}$ is given by the sum of $P_{mtd}$ and $P_{nmtd}$:

(5) $$P_{1}=\frac{1}{\delta }+\frac{\delta -1}{\delta }.$$

Likewise, total probability of second and third element of *S* and *T* is given by:

(6) $$P_{2}=\frac{1}{2\delta }+\frac{2\delta -1}{2\delta },$$

(7) $$P_{3}=\frac{1}{3\delta }+\frac{3\delta -1}{3\delta }.$$

Lastly, total probability of $n_{th}$ element of both data sets is:

(8) $$P_{n}=\frac{1}{n\delta }+\frac{n\delta -1}{n\delta }.$$

The average case complexity of the proposed sequential algorithm is:

(9) $$T_{avg}\left(ST\right)=\sum_{k=0}^{M}\sum_{i=0}^{m}\sum_{j=0}^{n}k\cdot i\cdot j\times P_{k}\cdot P_{i}\cdot P_{j}+\sum_{i=0}^{h}b\times p_{b},$$

putting values in Equation (9), we get:

(10) $$T_{avg}\left(ST\right)=M\cdot n\cdot \delta \cdot \left(\frac{1}{n}\ldots +\frac{1}{n}\right)\cdot \left(\frac{1}{n}\ldots +\frac{1}{n}\right)\cdot \left(\frac{1}{\delta }+\frac{\delta -1}{\delta }\ldots +\frac{1}{n\delta }+\frac{n\delta -1}{n\delta }\right)+h\cdot \left(\frac{1}{n}\ldots +\frac{1}{n}\right),$$

(11) $$T_{avg}\left(ST\right)=M\cdot n\cdot \delta \cdot \frac{1}{n}\left(1\ldots +1\right)\cdot \frac{1}{n}\left(1\ldots +1\right)\cdot \frac{1}{\delta }\left(1+\delta -1+\frac{1}{2}+\frac{2\delta -1}{2}\ldots +\frac{1}{n}+\frac{n\delta -1}{n}\right)+h\cdot \frac{1}{n}\left(1\ldots +1\right).$$

By applying algebraic manipulation, we get:

(12) $$T_{avg}\left(ST\right)=M\times n\times \delta +h.$$

### 5.2. Results and Discussion

**Figure 3:**
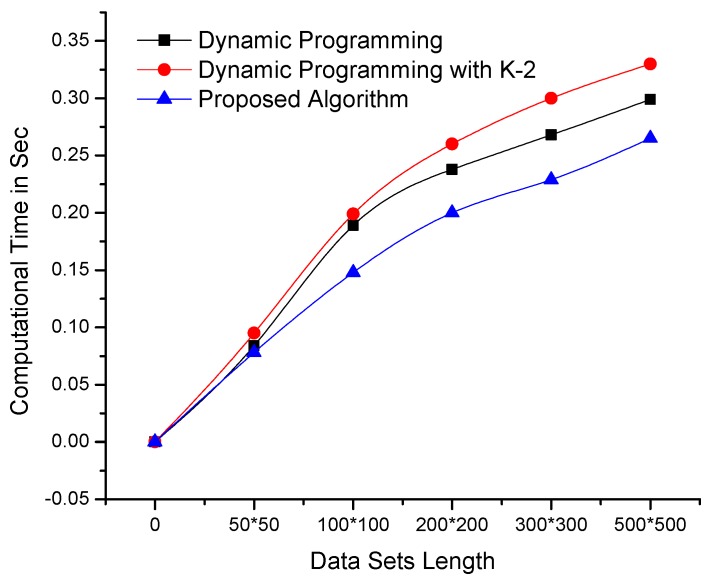
Comparison of the computational times of the proposed and dynamic programming-based algorithms—using data sets of constant length and variable dimensionality (the lowest lines i.e., minimum values are better).

The proposed algorithm and dynamic programming-based algorithms are implemented in a C++ development environment using similar resources and data sets. These algorithms were tested on benchmark data sets and a real-time data set obtained from our deployed WSN in Orange Orchard. Figure 3 clearly shows that the performance of the proposed algorithm is better than that of dynamic programming-based algorithms on data sets of constant length and dynamic variables or dimensions. Moreover, the proposed algorithm performs exceptionally well on data sets with high similarity indexes.

**Figure 4:**
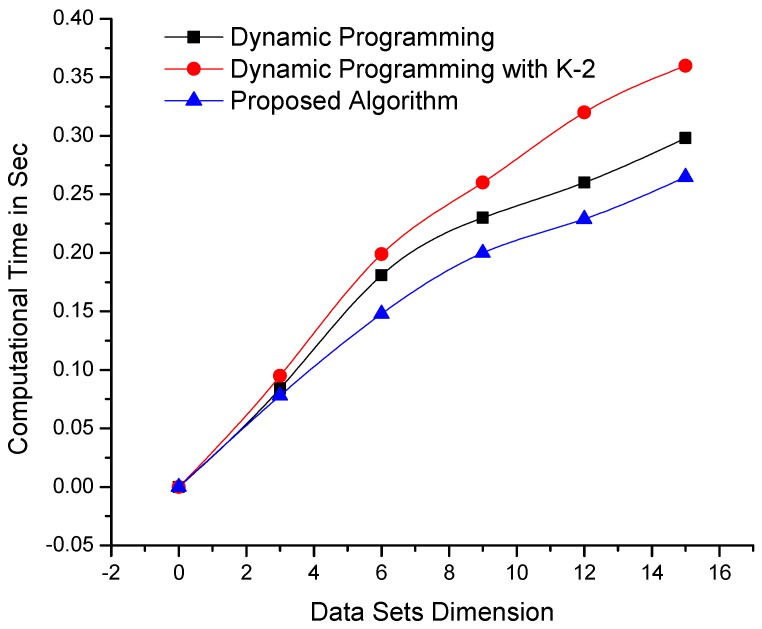
Comparison of the computational times of the proposed and dynamic programming-based algorithms—using data sets of variable length and constant dimensionality (the lowest lines i.e., minimum values are better).

**Figure 5:**
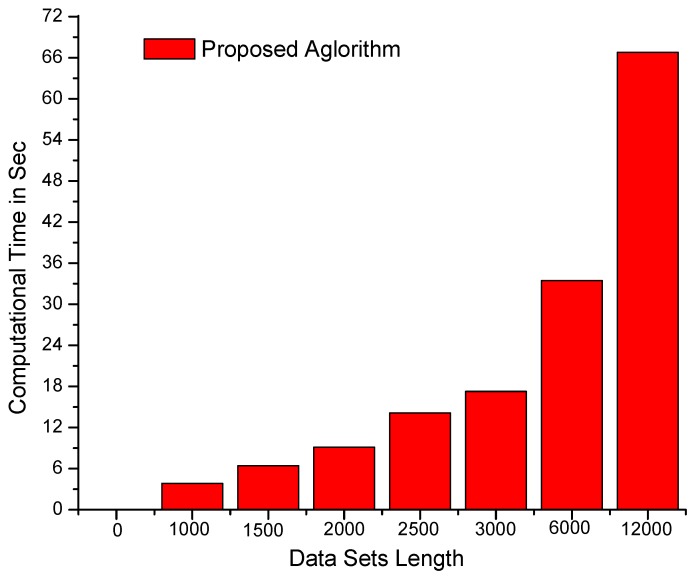
Computational time (in seconds) of the proposed algorithm on larger data sets (12,000 × 12,000 values) (the computational time increases linearly with respect to the size of data sets).

**Figure 6:**
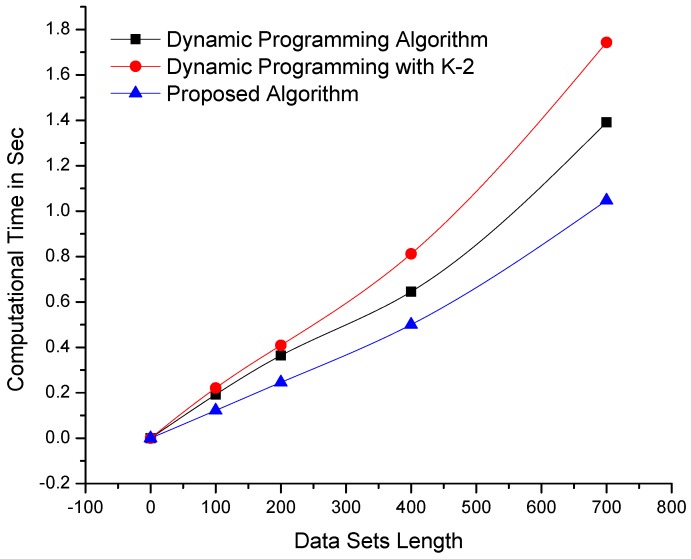
Comparison of the computational times of the proposed and dynamic programming-based algorithms using data sets of constant length and dimensionality. The lowest lines (i.e., the minimum values) reflect better performance.

The algorithms were also tested on data sets with constant variables/dimensions and dynamic sizes/lengths. The results of testing are shown in Figure 4, which clearly depicts the remarkable performance of the proposed algorithm compared with its counterpart algorithms. Although dynamic programming-based algorithms are affected by an increase in data set length or dimensionality, our mechanism is consistent. The algorithms were further tested on larger data sets with more complex dimensionalities, as shown in Figure 5, as well as on data sets where the parameters of length and dimensionality were kept constant. The computational results presented in Figure 6 demonstrate that the proposed algorithm performs better than dynamic programming-based algorithms.

Furthermore, the algorithms were tested on publicly available benchmark data sets obtained from NCBI viral genomes [31], data sets from the UCR time series repository [32], UCI (UCI KDD Archive, 2015) [33], the American Stock Exchange and the Onset Computer Corporation live data feeds (HOBO U30 Remote Monitoring Systems 2015–2016). The computational times presented in Table 3 reveal the consistency and superiority of the proposed algorithm over dynamic programming-based algorithms. The variations in computational time are largely due to the different dimensionalities of these data sets.

As shown in Table 3, the proposed algorithm performs approximately 5.7–33.4% more efficiently than DP-based algorithms for various benchmark data sets. Similarly, for DP-based algorithms with *k*-2, the proposed algorithm performs approximately 14.3–39.9% better. We observed that the proposed algorithm performs well for data sets with high dimensionality.

## 6. Conclusions and Future Works

LCSS is one of the most widely used mechanisms for determining the similarity indexes of different data sets, particularly univariate data sets, because non-metric-based approaches are insensitive to outliers where other mechanisms are. However, in the literature, these approaches are extensively studied in the context of univariate data sets; therefore, multivariate data sets remain an open research area because few studies on such data sets are available. In this study, we presented a computationally efficient non-metric-based algorithm to obtain the similarity of multivariate data sets using the sliding window concept; here, each symbol has a defined/fixed range of values to match with, which reduces not only the number of comparisons to be made but also the computational time and space requirements as opposed to traditional dynamic programming-based algorithms. The performance of the proposed algorithm was evaluated based on data sets of different sizes and dimensionalities, keeping in mind that real-time data sets are not consistently of the same size and dimensionality. The computational time of the proposed algorithm is exceptionally short in cases/data sets where the similarity index is extremely high. Due to the robustness of the proposed algorithm, it is suitable for use in different application areas, as confirmed by the results we obtained from dynamic, as well as publicly available, benchmark static data sets.

Our evaluation suggests that the proposed algorithm performs worst when multivariate data sets are completely different from each other, that is, no match can be found. Further research is needed to consider such scenarios and improve the performance of the proposed algorithm, particularly for large-scale data sets. Moreover, as of this writing, the sliding window-based control parameter is data-dependent. In the future, we aim to investigate how this dependency be resolved or at least minimised. An important application area of non-metric-based approaches is DNA comparison. An interesting research direction is implementation of the proposed technique for DNA comparison. Finally, integrating the proposed sliding window-based control parameter with traditional dynamic programming-based algorithms could improve the performance of the latter. Future research in this area may also be expected.

## Figures and Tables

**Table 1:** A short description of the collected time series data “S” for a single sensor node—measured at 15 min intervals (soil moisture represents soil wetness kilo ohm).

Date and Time	Temperature °C	Humidity %RH	Soil Moisture (kHz)
15 April 2011 22:00	35	92	780
15 April 2011 22:15	39	82	778
15 April 2011 22:30	36	87	776
15 April 2011 22:45	35	91	774
15 April 2011 23:00	37	85	772
15 April 2011 23:15	36	87	772
15 April 2011 23:30	38	83	770
15 April 2011 23:45	35	90	767
15 April 2011 00:00	38	82	762
15 April 2011 00:15	36	86	756

**Table 2:** A short description of the collected time series data “T” for a single sensor node—measured at 15 min intervals (soil moisture represents soil wetness kilo ohm).

Date and Time	Temperature °C	Humidity %RH	Soil Moisture (kHz)
16 April 2011 2:00	37	85	782
16 April 2011 2:15	35	92	780
16 April 2011 2:30	36	87	776
16 April 2011 2:45	38	84	775
16 April 2011 3:00	35	91	774
16 April 2011 3:15	37	85	772
16 April 2011 3:30	38	83	770
16 April 2011 3:45	35	90	767
17 April 2011 0:00	38	81	762
17 April 2011 0:15	36	86	756

**Table 3:** Comparison of the computational times (in seconds) of the proposed and dynamic programming-based algorithms—using benchmark data sets (minimum values, as shown in bold face, are better) [31,32,34].

Data Sets	Computational Time (Seconds)		
	DP-Based Algorithm	DP-Based Algorithm	Proposed Algorithm
		with k-2	
Hobolink-500	1.1091	1.1880	0.9220
Amex-500	0.625	0.8060	0.5470
Robotic-300	0.2810	0.3170	0.2650
Haptitus-500	0.4220	0.4561	0.3910
Twopattern-500	1.1720	1.2138	0.7810
Bacteria-700	1.3910	1.7430	1.0470

## References

[1] Polak A. (2018). Why is it hard to beat *O*(*n*^2^) for Longest Common Weakly Increasing Subsequence?. Inf. Process. Lett..

[2] Wang X., Mueen A., Ding H., Trajcevski G., Scheuermann P., Keogh E. (2013). Experimental comparison of representation methods and distance measures for time series data. Data Min. Knowl. Discov..

[3] Mikalsen K.Ø, Bianchi F.M., Soguero-Ruiz C., Jenssen R. (2018). Time series cluster kernel for learning similarities between multivariate time series with missing data. Pattern Recognit..

[4] Han J., Pei J., Kamber M. (2011). Data Mining: Concepts and Techniques.

[5] Tseng K.T., Chan D.S., Yang C.B., Lo S.F. (2018). Efficient merged longest common subsequence algorithms for similar sequences. Theor. Comput. Sci..

[6] Li Y., Li H., Duan T., Wang S., Wang Z., Cheng Y. A real linear and parallel multiple longest common subsequences (MLCS) algorithm. Proceedings of the 22nd ACM SIGKDD International Conference on Knowledge Discovery and Data Mining.

[7] Wang H. (2007). All Common Subsequences. IJCAI.

[8] Silva D.F., Giusti R., Keogh E., Batista G.E. (2018). Speeding up similarity search under dynamic time warping by pruning unpromising alignments. Data Min. Knowl. Discov..

[9] Chatfield C. (2018). Introduction to Multivariate Analysis.

[10] Breiman L. (2017). Classification and Regression Trees.

[11] Chiu B., Keogh E., Lonardi S. Probabilistic discovery of time series motifs. Proceedings of the Ninth ACM SIGKDD International Conference on Knowledge Discovery and Data Mining.

[12] Mueen A., Keogh E., Zhu Q., Cash S., Westover B. Exact discovery of time series motifs. Proceedings of the 9th SIAM International Conference on Data Mining.

[13] Lin X., Li Z. The similarity of multivariate time series and its application. Proceedings of the 4th International Conference on Management of e-Commerce and e-Government.

[14] Benson G., Levy A., Shalom B.R. Longest common subsequence in k length substrings. Proceedings of the 6th International Conference on Similarity Search and Applications.

[15] Deorowicz S., Grabowski S. (2014). Efficient algorithms for the longest common subsequence in k-length substrings. Inf. Process. Lett..

[16] Sadiq A.S., Alkazemi B., Mirjalili S., Ahmed N., Khan S., Ali I., Pathan A.S.K., Ghafoor K.Z. (2018). An Efficient IDS Using Hybrid Magnetic Swarm Optimization in WANETs. IEEE Access.

[17] Ueki Y., Hendrian D., Kurihara M., Matsuoka Y., Narisawa K., Yoshinaka R., Bannai H., Inenaga S., Shinohara A. Longest common subsequence in at least k length order-isomorphic substrings. Proceedings of the 43rd International Conference on Current Trends in Theory and Practice of Computer Science.

[18] Shahabi C., Yan D. Real-time Pattern Isolation and Recognition Over Immersive Sensor Data Streams. Proceedings of the MMM 2003 9th International Conference on Multi-Media Modeling.

[19] Keogh E., Chakrabarti K., Pazzani M., Mehrotra S. (2001). Locally adaptive dimensionality reduction for indexing large time series databases. ACM Sigmod Rec..

[20] Yang K., Shahabi C. A PCA-based similarity measure for multivariate time series. Proceedings of the 2nd ACM International Workshop on Multimedia Databases.

[21] Candès E.J., Li X., Ma Y., Wright J. (2011). Robust principal component analysis?. J. ACM.

[22] Vlachos M., Kollios G., Gunopulos D. Discovering similar multidimensional trajectories. Proceedings of the 18th International Conference on Data Engineering.

[23] Duchêne F., Garbay C., Rialle V. Similarity measure for heterogeneous multivariate time-series. Proceedings of the 12th European Signal Processing Conference.

[24] Apostolico A. (1997). String editing and longest common subsequences. Handbook of Formal Languages.

[25] Sakurai Y., Yoshikawa M., Faloutsos C. FTW: Fast similarity search under the time warping distance. Proceedings of the 24th ACM SIGMOD-SIGACT-SIGART Symposium on Principles of Database Systems.

[26] Rakthanmanon T., Campana B., Mueen A., Batista G., Westover B., Zhu Q., Zakaria J., Keogh E. Searching and mining trillions of time series subsequences under dynamic time warping. Proceedings of the 18th ACM SIGKDD International Conference on Knowledge Discovery and Data Mining.

[27] Gorecki T., Luczak M. (2015). Multivariate time series classification with parametric derivative dynamic time warping. Expert Syst. Appl..

[28] Shojafar M., Pooranian Z., Naranjo P.G.V., Baccarelli E. (2017). FLAPS: Bandwidth and delay-efficient distributed data searching in Fog-supported P2P content delivery networks. J. Supercomput..

[29] Ramírez-Gallego S., Krawczyk B., García S., Woźniak M., Herrera F. (2017). A survey on data preprocessing for data stream mining: Current status and future directions. Neurocomputing.

[30] Khan R., Ali I., Zakarya M., Ahmad M., Imran M., Shoaib M. (2018). Technology-Assisted Decision Support System for Efficient Water Utilization: A Real-Time Testbed for Irrigation Using Wireless Sensor Networks. IEEE Access.

[31] Coordinators N.R. (2016). Database resources of the national center for biotechnology information. Nucleic Acids Res..

[32] Dua D., Karra Taniskidou E. (2017). UCI Machine Learning Repository. http://archive.ics.uci.edu/ml.

[33] Chen Y., Keogh E., Hu B., Begum N., Bagnall A., Mueen A., Batista G. The UCR Time Series Classification Archive. http://www.cs.ucr.edu/~eamonn/time_series_data/.

[34] Bay S.D., Kibler D., Pazzani M.J., Smyth P. (2000). The UCI KDD archive of large data sets for data mining research and experimentation. ACM SIGKDD Explor. Newsl..

